# A Journey Under the Sea: The Quest for Marine Anti-Cancer Alkaloids

**DOI:** 10.3390/molecules16119665

**Published:** 2011-11-23

**Authors:** Rita Tohme, Nadine Darwiche, Hala Gali-Muhtasib

**Affiliations:** 1 Department of Biology, American University of Beirut, Beirut, P.O. Box 11-0236, Lebanon; Email: rgt07@aub.edu.lb; 2 Department of Biochemistry and Molecular Genetics, American University of Beirut, Beirut, P.O. Box 11-0236, Lebanon; Email: nd03@aub.edu.lb; 3 Department of Biology, American University of Beirut, Beirut, P.O. Box 11-0236, Lebanon

**Keywords:** alkaloid, marine, anti-cancer, drug, cell death

## Abstract

The alarming increase in the global cancer death toll has fueled the quest for new effective anti-tumor drugs thorough biological screening of both terrestrial and marine organisms. Several plant-derived alkaloids are leading drugs in the treatment of different types of cancer and many are now being tested in various phases of clinical trials. Recently, marine-derived alkaloids, isolated from aquatic fungi, cyanobacteria, sponges, algae, and tunicates, have been found to also exhibit various anti-cancer activities including anti-angiogenic, anti-proliferative, inhibition of topoisomerase activities and tubulin polymerization, and induction of apoptosis and cytotoxicity. Two tunicate-derived alkaloids, aplidin and trabectedin, offer promising drug profiles, and are currently in phase II clinical trials against several solid and hematologic tumors. This review sheds light on the rich array of anti-cancer alkaloids in the marine ecosystem and introduces the most investigated compounds and their mechanisms of action.

## 1. Introduction

Cancer is a devastating disease with tremendous negative implications at the personal, health care, economical and social levels. It is one of the leading causes of death in the World, afflicting an estimated 7.9 million people in 2007 (WHO data), and accounting for around 13% of all deaths worldwide. The search for an effective cure for cancer continues to improve patients’ survival. Although radiotherapy and surgery prevailed as treatment strategies at the beginning of the 20th century, the recovery rates never exceeded 33%, urging the need for an adjuvant therapy, namely chemotherapy [1]. The use of chemotherapy has resulted in major advances in the treatment of various types of cancer, when a large panel of compounds was screened on transplanted tumor systems to detect cytotoxic activities and regression of aberrant growths [1,2]. Large-scale screening programs endorsed by the US National Cancer Institute (NCI) led to the identification of promising anti-cancer products from various natural sources including plants, microbes, marine organisms, and animals [3].

The clinical use of natural products, especially terrestrial ones, is not recent as early medical scripts dating back to ancient Egypt describe the usage of plants to treat malignancies [3]. However, the later development of such natural products relied on biotechnological advances and novel approaches such as high-throughput screening and combinatorial synthesis [4]. Although marine-derived compounds are the least represented among the natural products, marine life is becoming increasingly exploited as scientists are overcoming limitations related to accessibility of aquatic samples [5]. The oceans, which offer a biologically rich ecosystem that covers 70% of the Earth, contain a large number of organisms which produce an array of compounds to help them withstand extreme conditions of temperature and pressure and to provide protection against predators [6]. One class of these compounds is the nitrogen-containing alkaloids. Plant-derived alkaloids such as vinca alkaloids have already proven their efficacy in treating tumors and have been leading anti-cancer drugs [3].

Screening of cyanobacteria, fungi, sponges, algae, and tunicates has led to the identification of a number of anti-cancer alkaloids. However, the difficulties of developing drugs from marine sources have resulted in their relatively low numbers in clinical trials or in clinical use [4]. Even with these difficulties, the two alkaloids aplidin and trabectedin have reached phase II clinical trials for the treatment of many solid and hematologic tumors. In this review, we focus on the latest and most prominently researched marine alkaloids in the cancer field. We also discuss the mode of cell death in various tumor cells with a focus on their molecular mechanism of action.

## 2. Anti-Cancer Alkaloids Derived from Microorganisms

### 2.1. Cyanobacteria: A Pool of Bioactive Alkaloids

The fact that cyanobacteria undergo photosynthesis and fix nitrogen allows these microorganisms to form large colonies. This feature makes it possible to investigate the pharmacological and genetic characteristics of cyanobacteria. Interestingly, their prominent presence subjects them to predation which necessitates the production of poisonous chemicals in order for them to survive [7].

The rich array of bioactive chemicals isolated from cyanobacteria made this group an interesting target for further investigations. Out of the 800 compounds extracted from marine cyanobacteria, 300 alkaloids have been reported, with the majority isolated from the genera *Lyngbya* and *Symploca* [8,9]. These marine alkaloids are mostly produced as potent toxins against predators. They consist of secondary metabolites with unusual structures and diverse range of activities against various diseases, including cancer. Investigations are being undertaken to determine the structures, biosynthetic pathways, and mechanisms of actions of these alkaloids with the hope of developing novel drugs to fight cancer [7,8,9].

**Figure 1:**
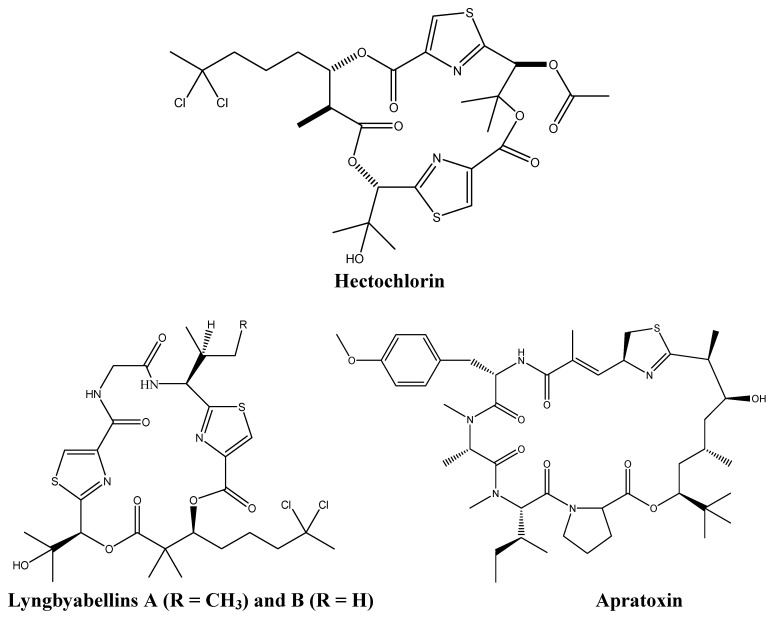
Chemical structures of hectochlorin, lyngyabellins, and apratoxin.

Around 30% of the bioactive molecules derived from cyanobacteria are synthesized by the marine filamentous blue green algae *Lyngbya majuscula* [7]*.* The most important alkaloids produced by this organism are hectochlorin, and the lyngyabellins which target actin or tubulin polymerization, and, the apratoxins, which exhibits high levels of cytotoxicity (Figure 1).

Both the cyclic depsipeptides hectochlorin and lyngyabellin, target the actin filaments of the cells. Hectochlorin, which causes hyperpolymerization of actin proteins, induces G_2_/M phase arrest in human Burkitt lymphoma CA46 cells. Cytotoxic testing of this metabolite against 60 cancer cell lines showed its remarkable anti-cancer effects on colon, melanoma, ovarian, and renal cancers [10,11].

Like hectochlorin, the lyngyabellins displayed anti-proliferative mechanisms in various cell types. Lyngyabellins E, D, F and H exhibited high levels of toxicity against the human lung cancer cell line NCIH460, while lyngyabellin I was cytotoxic against the mouse neuroblastoma neuro2-a cells [9,11,12]. Both acyclic and cyclic lyngyabellins were active *in vitro*, raising questions concerning possible alternations between the two conformations to increase bioavailability [12]. The most extensively studied compound, lyngyabellin A, was shown to disrupt the microfilament network, and accordingly to disrupt cytokinesis in colon carcinoma cells causing the formation of apoptotic bodies [12,13,14]. Lyngyabellin B is believed to act in a similar fashion to lyngyabellin A and its biological activity is under recent investigation [15,16]. Most importantly, the specificity of these lyngyabellins to cancer cells renders them attractive potential anti-cancer drugs [9].

The cyclic depsipeptides apratoxin A and B showed promising *in vitro* anti-cancer activities against many human tumor cell lines including LoVo colon cancer cells and nasopharynx human carcinoma KB cells. However, these alkaloids did not have any anti-cancer *in vivo* effects in colon or mammary cancers, and caused severe side effects in treated mice [17,18]. Investigation of the mechanism of action of apratoxin has shown that this drug causes mitotic cell cycle arrest at G_1_ phase leading to apoptosis [19]. One mode of apoptosis induction by apratoxin is blocking the fibroblast growth factor receptor (FGFR) pathway via the inhibition of the phosphorylation of the signal transducer and activator of transcription 3 (STAT3) in a time- and dose-dependent manner, thus inhibiting the upregulation of anti-apoptotic genes like Bcl_xL_ and the activation of the cyclin-dependent kinase Cdk2 [19]. These effects may explain apratoxin’s anti-angiogenic effects due to its ability to antagonize the FGFR pathway [19].

**Figure 2:**
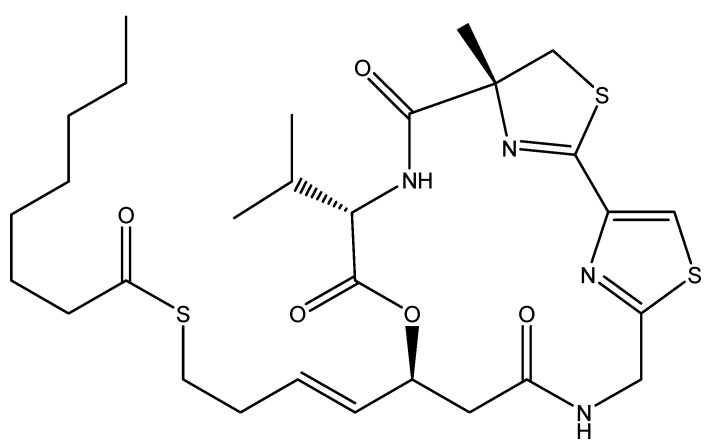
Chemical structure of largazole.

Screening of metabolites derived from *Lyngbya symploca* led to the discovery and structure elucidation of the promising anti-cancer agent largazole (Figure 2). Largazole is a macrocyclic depsipeptide that exhibits a selectively potent anti-cancer activity through the inhibition of histone deacetylases (HDAC) [20]. Since the normal biological activity of HDAC is involved in regulating transcription in eukaryotic cells, HDAC inhibitors will induce cell differentiation and cell death, thus blocking the proliferation of tumor cells [20]. This renders HDAC inhibitors such as largazole promising anti-cancer drug candidates [21].

### 2.2. New Alkaloids from Marine Fungi: An Embryonic Stage

The unique array of secondary metabolites produced by marine fungi is mainly due to the extreme conditions in which fungal species grow and to several symbiotic relationships they undergo with other marine organisms [22]. Marine fungi live under different levels of pressure, temperature, salinity and nutrient availability than their terrestrial counterparts which led to the development of novel biological pathways and the production of new molecules that enhance their adaptation to harsh conditions [23].

Screening for the anti-cancer potential of marine fungi metabolites revealed that many had promising anti-tumorigenic properties against numerous human cancer cell lines. The most studied alkaloids were extracted from *Aspergillus*, *Penicillium* and *Actinomycetes* species, commonly via bioassay-guided fractionation. Although some of the fungi-derived alkaloids introduced in this review induce apoptosis, and others show cytotoxicity and antioxidant activities, the mechanism of action of these drugs is yet to be elucidated.

**Figure 3:**
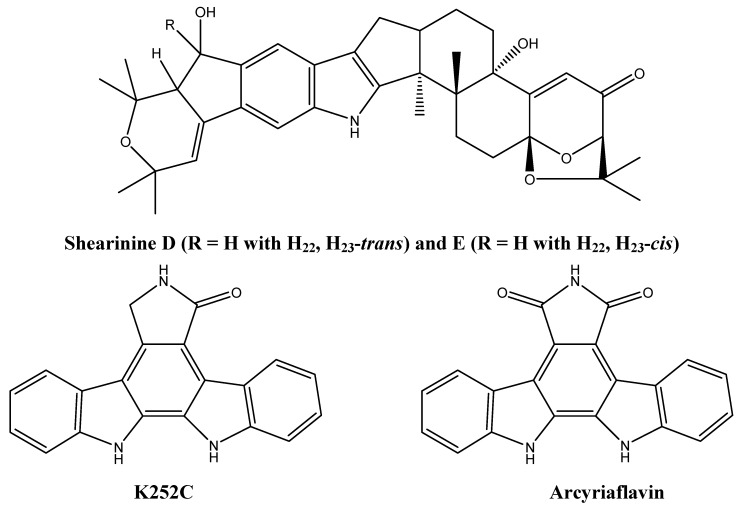
Chemical structures of shearinine D and E, K252C, and arcyriaflavin.

Alkaloids shown to cause apoptosis in cancer cells were isolated from *Penicillium janthinellum* and Actinomycete Z_2_039-2 [24,25]. Three indole alkaloids, shearinine A, D, and E (Figure 3), were isolated from *Penicillium janthinellum* and were shown to induce apoptosis in human promyelocytic leukemia cells HL-60 [25]. Furthermore, shearinine E inhibits the malignant transformation of mouse epidermal JB6P+ cells into malignant cells [25]. This JB6P+ transformation occurs in an endothelial growth factor (EGF)-dependent manner and shearinine E is thought to inhibit EGF action [25]. Two indolocarbazole alkaloids (K252C and arcyriaflavin, Figure 3) were isolated from Actinomycete Z_2_039-2 via bioassay-guided fractionation and were shown to induce apoptosis in the myelogenous leukemia cell line (K562) [24].

**Figure 4:**
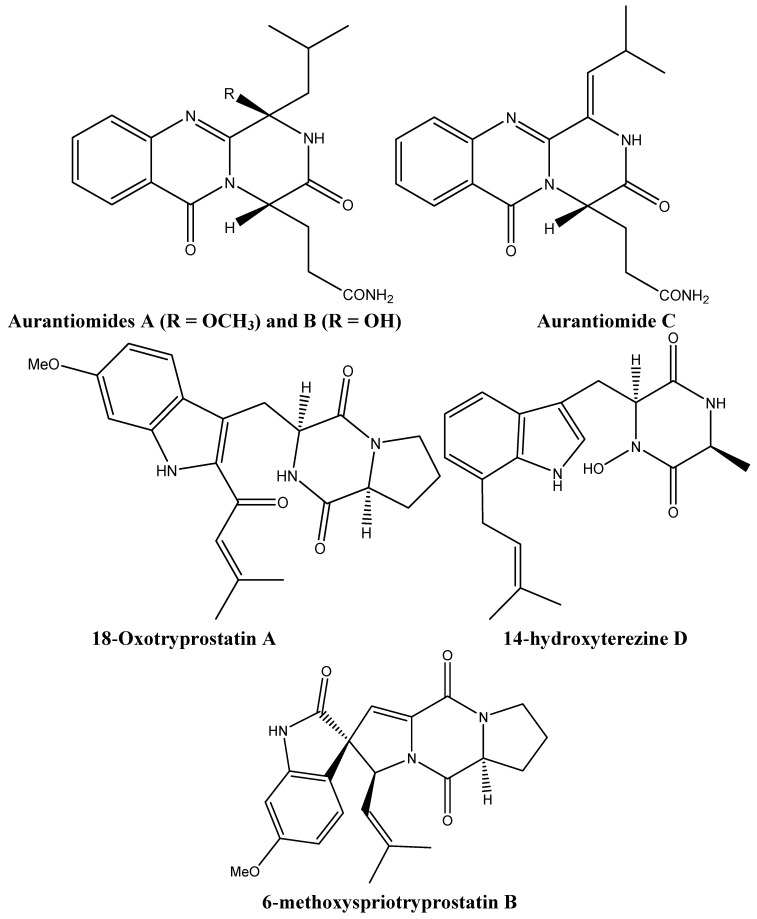
Chemical structures of aurantiomides A, B, and C, 18-Oxotryprostatin A, 14-hydroxyterezine D, and 6-methoxyspriotryprostatin B.

Metabolites derived from *Penicillium aurantiogriseum* and *Aspergillus sydowi* were cytotoxic to several tumor cells. The three quinazoline alkaloids, aurantiomides A, B, and C (Figure 4) were derived from *Penicillium aurantiogriseum;* only the latter two were found to be cytotoxic against the lymphoblastic P388 and HL-60 cell lines (aurantiomide B) and human hepatoma cells BEL-7402 and P388 (aurantiomide C) [26]. 18-Oxotryprostatin A, 14-hydroxyterezine D, and 6-methoxyspriotryprostatin B (Figure 4) were isolated from *Aspergillus sydowi* and have shown weak cytotoxicity against human alveolar basal carcinoma A-549 cells. In addition, 6-methoxyspriotryprostatin B (Figure 4) was active against HL-60 cells [27].

## 3. Sponge-Derived Alkaloids: Molecules with Defined Mechanisms of Action

**Figure 5:**
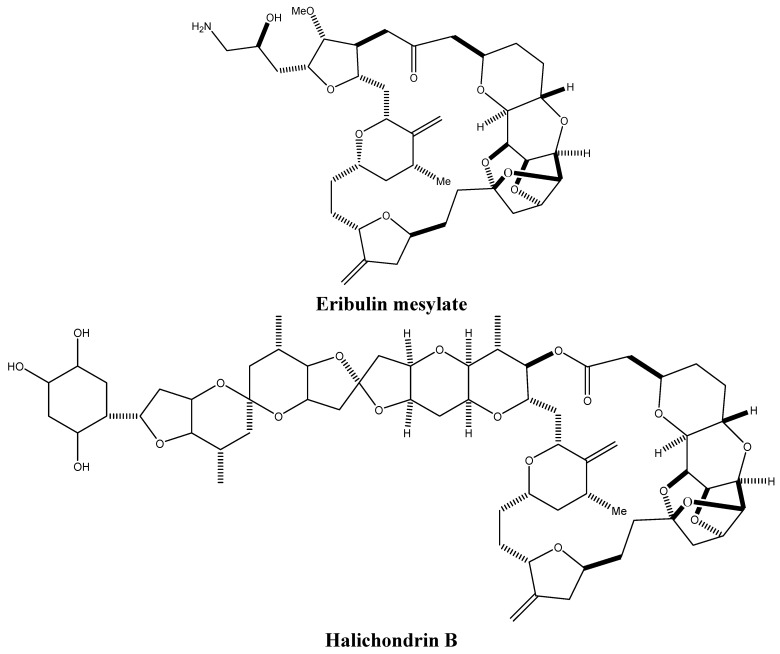
Chemical structures of eribulin mesylate and halichondrin B.

Marine sponges, which mostly inhabit salty water, evolved a long time ago and are considered as one of the oldest life forms. Although the secondary metabolites produced by these marine animals are relatively low in concentrations, they help them deter predators and compete with sessile species [28]. Furthermore, sponges undergo symbiotic relationships with some microorganisms such as bacteria and fungi, which are likely to be the ones supplying the bioactive molecules [29,30]. More than 10% of the screened sponges display cytotoxic activities [31,32,33], and a number of the identified compounds and their analogs have reached clinical trials such as eribulin mesylate (Figure 5), an analog of the macrocyclic polyether halichondrin B (Figure 5), which reached phase I and II cancer clinical trials for the treatment of metastatic breast cancer [34]. Unlike other marine-derived alkaloids, the mechanisms of action of many sponge-derived alkaloids have been elucidated and many exhibit actions similar to the clinically used plant-derived chemotherapeutic drugs paclitaxel and camptothecins. These marine-derived alkaloids inhibit cell proliferation through targeting topoisomerases and tubulin polymerization, and modulate the balance of anti-apoptotic and pro-apoptotic proteins. Some are even able to restrain cell migration and invasion, thereby hindering metastasis.

### 3.1. Targeting Tubulin Polymerization

Tubulins are major pillars of the cytoskeleton organization and play a central role in mitotic division, cellular migration, and intracellular trafficking of vesicles and organelles [35]. Since cancer cells divide at a higher rate than normal cells, they are the targets of choice for anti-mitotic drugs [36,37]. Anti-cancer drugs targeting microtubule polymerization or depolymerization, such as taxol and vinca alkaloids have become the most effective mitotic inhibitors.

**Figure 6:**
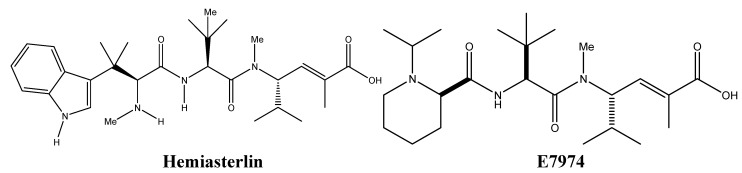
Chemical structures of hemiasterlin and E7974.

Hemiasterlin and E7974 (a hemiasterlin synthetic analogue) (Figure 6) are sponge-derived alkaloids that possess tubulin-based antimitotic mechanisms. The peptides hemiasterlins A and B were originally derived from the marine sponge *Hemiasterella minor* and exhibit anti-proliferative effects against murine leukemia p388 cells, human glioblastoma U373 cells, human mammary carcinoma MCF-7 cells, and human ovarian carcinoma HEY cells [38,39]. Both hemiasterlins A and B bind to tubulin at nanomolar concentrations and inhibit their polymerization, causing mitotic catastrophe and ultimately apoptosis [40]. This mechanism is similar to that of vinca alkaloids, although hemiasterlin A does not produce vinblastine paracrystals nor microtubule bundles at higher concentrations [40,41]. Nevertheless, the *in vivo* effect of hemiasterlins was accompanied with a high level of toxicity, necessitating the synthesis of new analogues with enhanced drug profiles [38].

E7974 retains both the *in vitro* and *in vivo* antimitotic effects of hemiasterlin at nanomolar concentrations [42]. Cancer cells treated with E7974 are blocked at the G_2_/M phase of mitosis a few hours after drug exposure, creating a hypodiploid population of cells that finally undergoes programmed cell death via apoptosis [43]. E7974 induced apoptosis is associated with caspase 3 and poly ADP-ribose polymerase (PARP) cleavage as well as Bcl-2 phosphorylation [43]. The anti-mitotic effect of E7974 has been shown to be anchorage-independent since treating monolayer-growing human prostate cancer DU145 cells gave similar results to suspension grown human leukemic monocyte lymphoma U-937 cells [43]. E7974 directly targets microtubules similar to taxanes and vinca alkaloids. It binds selectively to the α-tubulin and to a lesser extent to the α/β-tubulin interface, and causes the disruption of the mitotic spindle formation and a decrease in the microtubule density [43]. E7974 optimal effect is observed prior to the decrease of the drug’s availability in the blood circulation between successive administrations [43]. Most interestingly, E7974 retains its effect in tumors that are resistant to taxanes and vinca alkaloids [42]. These resistant cells usually overexpress the drug efflux pump P-glycoprotein (Pgp) or have mutated β-tubulin on which taxanes and vinca alkaloids exert their action [42]. The ability of E7974 to overcome these limitations, as well as its efficacy in inhibiting tumor growth *in vivo*, advocates for its selection in human clinical trials [42].

### 3.2. Inhibiting Topoisomerases

Topoisomerases have been identified as targets of many anti-cancer drugs due to their crucial role in many cellular mechanisms such as DNA replication, transcription, recombination, and repair [44,45]. Topoisomerase II (TOP2) binds to both DNA strands, forming a reversible topoisomerase II cleavage complex (TOP2cc) and subsequently breaking the strands. This strand break relaxes the negative and positive supercoiling of the DNA strands and this is usually followed by religation by the TOP2 enzyme [46]. If TOP2 is unable to detach from the DNA, this will lead to double strands breaks inducing apoptosis [46]. Many anti-cancer drugs are topoisomerase inhibitors, such as the sponge-derived alkaloids neoamphimedine and makaluvamines, which bind to TOP2cc and inhibit its degradation [47].

**Figure 7:**
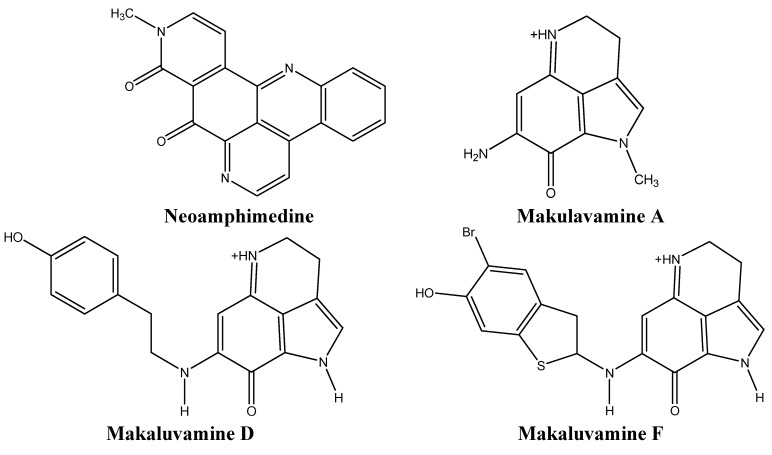
Chemical structures of neoamphimedine and makulavamines A, D, and F.

The pyridoacridine neoamphimedine (Figure 7) was isolated from *Xestospongia* and has a unique mechanism of action as a topoisomerase inhibitor [48]. Although this alkaloid exhibits an enhanced activity when cells overexpress TOP2, its level of toxicity is not affected in cells that lack this enzyme [49]. Contrary to other TOP2-dependent drugs, neoamphimedine’s mode of action is not only based on stabilizing the level of TOP2cc to induce DNA double strand breaks [49]. It also induces a TOP2-dependent catenation of the DNA *in vitro*, a characteristic that is found in other drugs such as spermidine, and polyethylene glycol. However, unlike neoamphimedine, classical TOP2 inhibitors are either needed at high concentrations, or have a low bioavailability [50,51]. This is why neoamphimedine has an advantage over currently used TOP2 inhibitors. Neoamphimedine showed similar efficacy to the clinically used TOP2 inhibitor etoposide in the *in vivo* xenograft models of nasopharynx human carcinoma KB and colorectal cancer HCT-116 cell lines injected in nude mice [49].

The makaluvamines (Figure 7) consist of a group of pyrroloiminoquinone alkaloids isolated from *Zyzzyac marsailis*, *Histodermella* sp., *Zyzzya fuliginosa*, and *Smenospongia aurea* [52,53,54,55]. Five of these alkaloids have been found to be highly cytotoxic to HCT-116 human colon cancer cells and MCF-7 and MDA-MB-468 human breast cancer cells via TOP2 inhibition mechanisms [56]. These alkaloids alter cellular mechanisms by causing double strand breaks in the DNA, and are therefore highly promising anti-cancer agents.

### 3.3. Targeting Molecular Players in Programmed Cell Death

**Figure 8:**
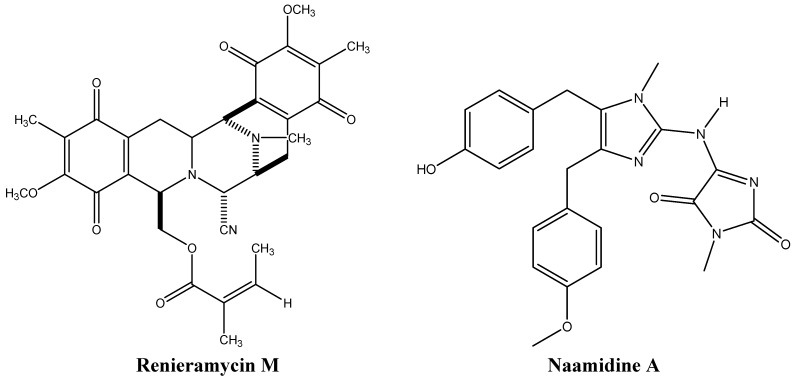
Chemical structures of renieramycin M and naamidine A.

Resistance to apoptosis is one of the hallmarks of cancer. Thus, anti-cancer drugs inducing apoptosis are considered as promising treatments. Some sponge-derived alkaloids, such as renieramycin M and naamidine A (Figure 8), target proteins that are involved in apoptosis induction.

Micromolar concentrations of the *Xestospongia* sponge isoquinoline renieramycin M, induce apoptosis in human non-small cell lung cancer H460 cells *in vitro* in a p53-dependent and anchorage-independent manner [57]. Treatment of H460 cells with renieramycin M was found to stabilize p53 and decrease the levels of the anti-apoptotic proteins Mcl-1 and Bcl-2 [57], suggesting that this molecule is a promising anti-cancer drug candidate, particularly for lung cancer treatment since Bcl-2 is overexpressed in these cancer cells [57,58,59].

Renieramycin M also targets the invasion and metastasis of tumor cells and sensitizes them to anoikis [57]. Indeed, resistance to anoikis allows cells to metastasize and survive after detachment. The renieramycin family of compounds was found to be highly cytotoxic to DLD1 human colon, HCT116 human colon, NCI-H460 human non-small cell lung, QC56 human lung, MDA-MB-435 breast, T47D human ductal breast epithelial, MDA-MB-435 breast, and AsPC1 human pancreatic cancer cell lines [58].

Naamidine, an imidazole identified in *Leucetta chagosensis*, is another example of an apoptosis-targeting sponge-derived alkaloid [60]. After determining the potency of this alkaloid in rodent tumor xenografts, its mode of action was deciphered [61]. Naamidine induces caspase-dependent apoptosis in tumors and immortalized cells via both extrinsic and intrinsic cell death pathways, accompanied by disruption of mitochondrial potential [61]. Naamidine provokes cell cycle arrest at G_1_ phase throughstabilization of the p21 cyclin-dependent kinase inhibitor (CDK2), activation of the initiating caspases 8 and 9, and PARP cleavage [61]. The effector caspase 3, common to both intrinsic and extrinsic pathways, is also cleaved by naamidine *in vitro* and in tumor xenografts *in vivo* [61]. Interestingly, this mechanism is p53-independent, which is of clinical relevance since 50% of cancers harbor p53 mutations [61,62].

### 3.4. Deregulating Cell Proliferation and Cell Cycle Control

**Figure 9:**
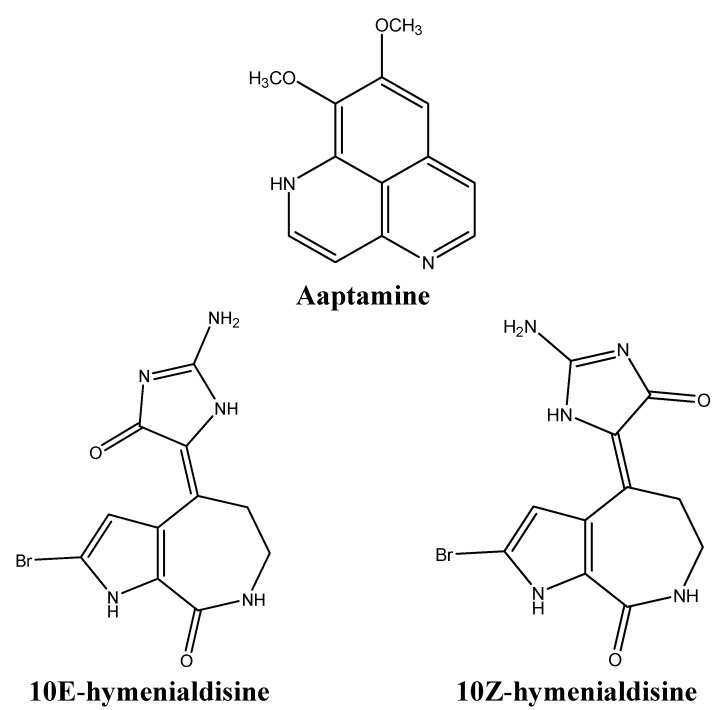
Chemical structures of aaptamine, 10E-hymenialdisine, and 10Z-hymenialdisine.

The rapid proliferation of cancer cells is a direct consequence of the imbalance between CDK-cyclin complexes and CDK inhibitors (CDKI) [63]. The CDK-cyclin complex inhibitor p21 is a downstream effector of the tumor suppressor p53 [64,65]. During cellular stress, p53 stabilization induces the expression of p21, which then acts as a negative regulator of the cell cycle. If the damage is repaired, the cell cycle continues, otherwise apoptosis follows. The mutation of p21 is rare in human tumors, making p21 an interesting target for “gene-regulating chemotherapy or prevention” [66]. The benzonaphthyridine alkaloid aaptamine (Figure 9) was extracted from the marine sponge *Aaptos suberitoides* in an attempt to isolate agents that would activate the promoter of p21 in a p53-independent manner [66]. Aaptamine was found to up-regulate p21 transcription in p53-mutated human osteosarcoma MG63, inducing G_2_/M cell cycle arrest [66]. Since 50% of malignancies harbor mutated p53, drugs like aaptamine offer new means to trigger apoptosis in a p53-independent fashion [65].

Aldisine alkaloids, isolated from the Philippine sponge *Stylissa massa* (Figure 9) are potent inhibitors of the mitogen-activated protein kinase kinase-1 (MEK-1) [67]. The cascade involving Raf/MEK/MAPK (mitogen activated MAP kinase) proteins is deeply involved in cellular signaling processes, ultimately leading to the phosphorylation of modulators of cell proliferation and differentiation [67]. Since oncogenic Ras signals through Raf/MEK/MAPK and is implicated in 30% of all cancers, targeting this oncogene is of high pharmacological importance [67]. Bioassay guided fractionation of *Stylissa massa* sponge extracts yielded two aldisine alkaloids, 10E-hymenialdisine and 10Z-hymenialdisine (Figure 9), that selectively inhibit the phosphorylation of MAPK by MEK-1, thus blocking the Raf/MEK/MAPK cascade [67]. Hymenialdisine also inhibits CDK1 [68].

**Figure 10:**
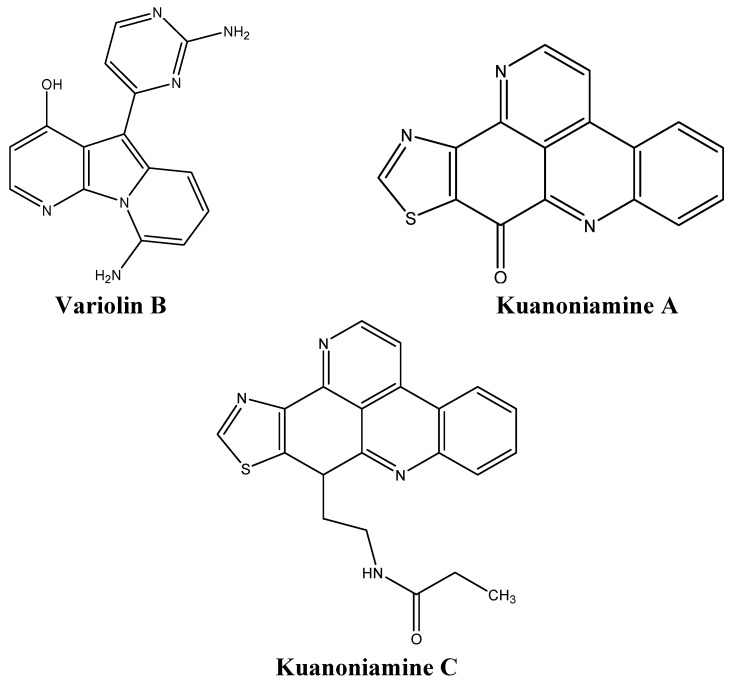
Chemical structures of variolin B, and kuanoniamines A and C.

Variolin B (Figure 10) a pyridopyrrolopyrimidine extracted from *Kirkpatrickia variolosa*, is cytotoxic against several human cancer cells, including human leukemia cells, ovarian, and intestinal carcinomas [69]. This alkaloid is also active in colon LoVo cells which are usually resistant to conventional chemotherapeutic agents due to high levels of Pgp expression [69]. Variolin B induces G_1_ arrest at nanomolar concentrations and G_2_ arrest at micromolar concentrations, and subsequent apoptosis at both concentrations [69].

The proliferation of five human cancer cell lines was tested after treatment with the two alkaloids derived from *Oceanapia sagittaria*, namely kuanoniamines A and C (Figure 10) [70]. Although kuanoniamine A was more potent than kuanoniamine C, the latter was more specific to estrogen dependent breast cancer cells [70]. Kuanoniamine A causes a decrease in cells in the S and G_2_/M phases, as well as an accumulation of cells in the G_1_ phase [70].

### 3.5. Targeting Angiogenesis

The pathogenesis of malignancies resides in the ability of tumors to induce the development of new blood vessels from pre-existing ones, a process termed angiogenesis [71]. Targeting this process and inhibiting the main pro-angiogenic player, the vascular endothelial growth factor (VEGF), is crucial for therapeutic interventions that aim at preventing tumor growth and inhibiting metastasis [71]. Several sponge-derived alkaloids target various tumor invasion processes such as proliferation, migration and tubular formation of endothelial cells [72].

**Figure 11:**
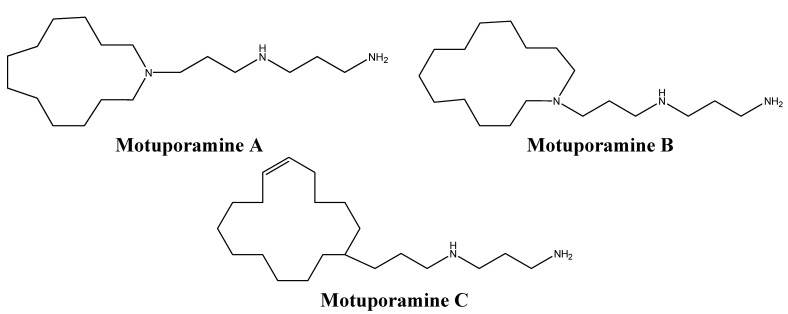
Chemical structures of motuporamines A, B, and C.

Bioassay guided-fractionation of *Xestospongia exigua* extracts led to the identification of a family of anti-invasive and anti-angiogenic alkaloids, particularly the motuporamines A, B, and C (Figure 11) [73,74]. Motuporamines inhibited *in vitro* invasion of basement membranes by many tumors cells such as MDA-231 breast carcinoma and PC-3 prostate carcinoma cells [74]. Motuporamine C, the most potent motuporamine among these molecules, induces cytoskeletal changes in cancer cells, hinders the activation of β1-integrin, which plays a crucial role in adhesion and invasion of cancer cells, and inhibits cell migration and angiogenesis [74]. Although motuporamine C does not alter cell proliferation, the fact that it targets steps of the invasion-metastatic cascade and is easy to synthesize makes it a promising potential anti-cancer drug [75].

**Figure 12:**
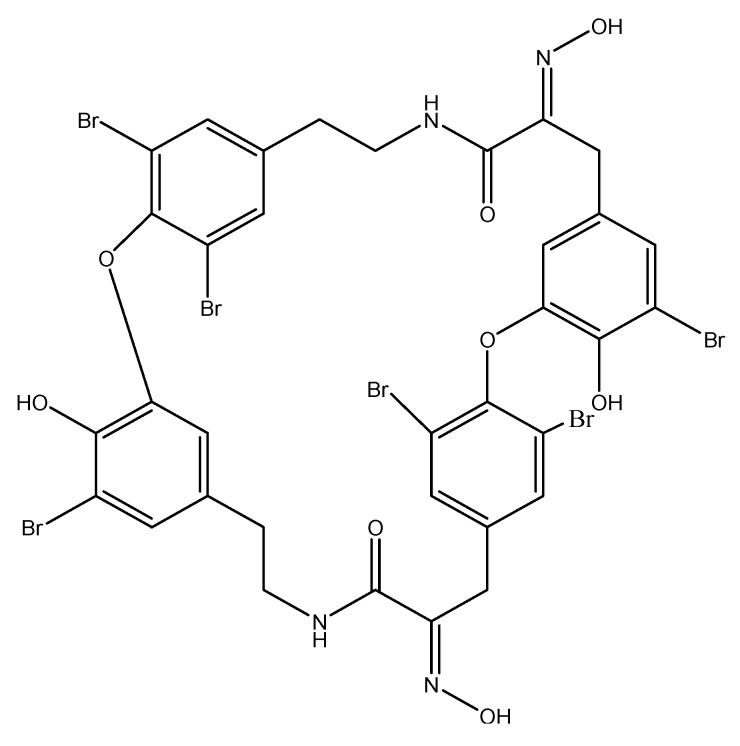
Chemical structure of bastadin 6.

Another interesting sponge-derived alkaloid that targets angiogenesis is the macrocyclic tetramer bastadin 6 (Figure 12), isolated from the *Dendrilla cactos* sponge. When bastadin 6 was added to human umbilical vein endothelial cells, it inhibited VEGF and basic fibroblast growth factor (bFGF)-dependent proliferation [72], two factors that play a crucial role in tumor angiogenesis.

### 3.6. Targeting Isoprenylcysteine Carboxyl Methyltransferase

**Figure 13:**
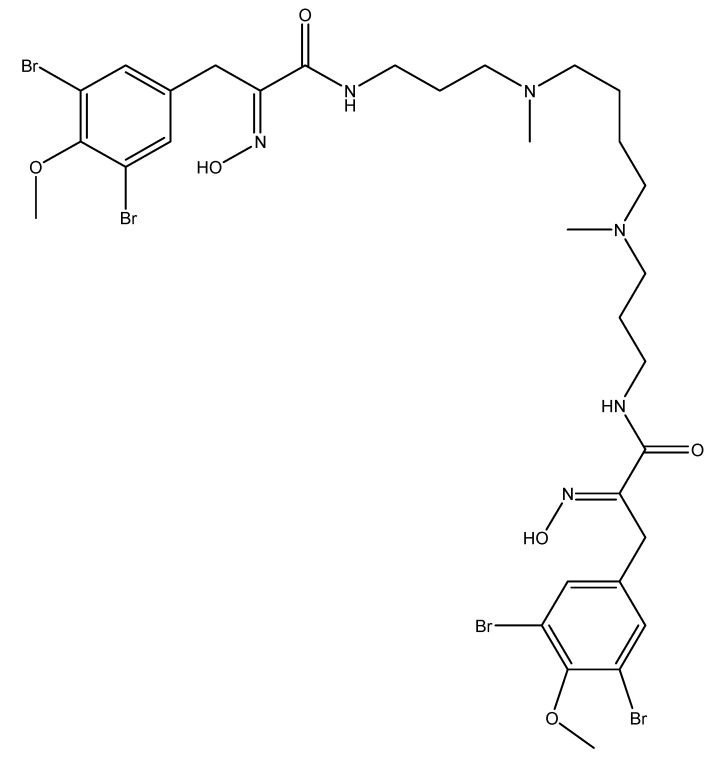
Chemical structure of spermatinamine.

Many key proteins that are important in oncogenesis, including the members of the Ras family of GTPases, are modified at their C terminus by a process called prenylation. This process involves the addition of an isoprenoid lipid followed by further modification by proteolysis and methylation of the C-terminal prenylcysteine. These posttranslational modifications are important for protein localization and function. The enzyme isoprenylcysteine carboxyl methyltransferase catalyzes the carboxyl methylation of Ras and several other oncogenic proteins as a last step of a series of post-translational modiﬁcations [77]. Recent studies have provided strong evidence that blocking the activity of isoprenylcysteine carboxyl methyltransferase by genetic disruption has profound consequences on oncogenic transformation and limits the proliferation of cancer cells, thus rendering this enzyme an attractive target for anti-cancer drugs [76,77]. A high-throughput screening led to the identification of the alkaloid spermatinamine (Figure 13), derived from the Australian marine sponge, *Pseudoceratina* [76]. Spermatinamine is the first known drug to inhibit the activity of the enzyme isoprenylcysteine carboxyl methyltransferase.

### 3.7. Cytotoxic Sponge-Derived Alkaloids with No Defined Mechanisms

Many sponge-derived alkaloids have been screened for their anti-cancer effect and exhibit potent cytotoxic activities. However, their mechanisms of action have not been investigated yet. These alkaloids are summarized in Table 1 along with their origin, the tumor cell lines they affect, and the concentration range which causes 50% reduction in cell viability (IC_50_).

**Table 1:** Cytotoxic anti-cancer sponge-derived alkaloids.

Alkaloid	Organism	Cell Lines	IC_50_ (μg/mL)	References
Amphimedine	*Amphimedon* sp.	U-87MG, U-373Mg, J82, HCT15, LoVo, A549	0.1–3.1	[78]
Arenosclerin A–C	*Arenosclera brasiliensis*	HL60, B16, U138, L929	<5	[79]
Echinoclathrines A–C	*Echinoclathria* sp.	P388, A549m HT29	10	[79]
Haliclonacyclamie E	*Arenosclera brasiliensis*	HL60, B16, U138, L929	<5	[79]
Halitulin	*Haliclona tulearensis*	P388, A549, MEL28	0.012–0.025	[80]
Longamide	*Agelas longissima, Homaxinella*	P388	Not determined	[81,82]
Ma’edamines A and B	*Suberea* sp.	L1210, KB	3.9–5.2	[83]
Matemone	*Iotrochota purpurea*	NSCLC-N6 L16, Mia PaCa-2, DU145	24–30	[84]
*N-*Methyl-*epi*-manzamine D, *epi*-Manzamine D	PAL93055	B16F10	0.1	[85]
Nortopsentins A, B, and C	*Spongsorites ruetzleri*	P388	1.7–7.8	[86]
Pyrinodemin A-D	*Amphimedon* sp.	L1210, KB	0.06–0.08	[87,88]
Topsentin B1 and B2	*Rhaphisia lacazei*	NSCLC-N6	6.3–12	[89]

## 4. Tunicate-Derived Anti-Cancer Alkaloids: The Future Promise

Traditionally, tunicates are known to be a major source of biomedical compounds for cancer treatment [90]. In addition to aplidin and trabectedin, other alkaloids isolated from tunicates have been identified as promising new anti-cancer drugs.

### 4.1. Aplidin: Oxidative-Stress Mediated Apoptosis

**Figure 14:**
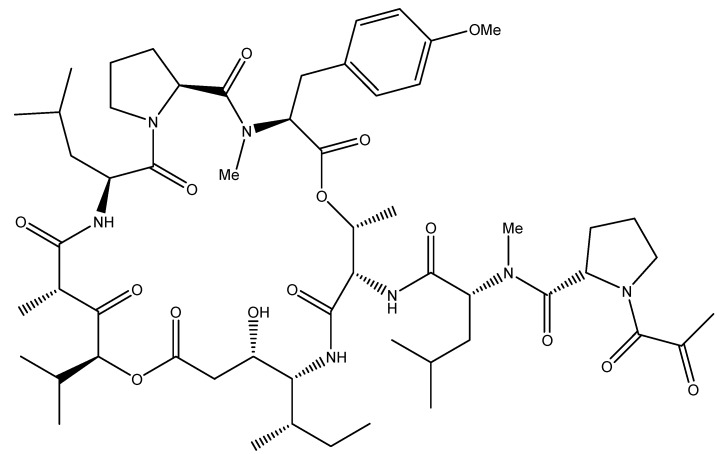
Chemical structure of tunicate-derived drug aplidine.

The potent anti-neoplastic drug aplidin (Figure 14), or plitidepsin, was isolated from the Mediterranean marine tunicate *Aplidium albicans* and is currently in phase II clinical trials against various hematological and solid tumors such as melanoma, multiple myeloma, peripheral T-cell lymphoma, and metastatic clear cell renal carcinoma [91,92]. Depending on the cancer cell type, this cyclic depsipeptide can either induce stress-mediated apoptosis, hinder the cell cycle transition from S to G_2_ and/or block it in G_1_, independently of the cells’ p53 tumor suppressor status [69,93].

Aplidin induces an early oxidative stress response in cancer cells as it alters glutathione homeostasis [94]. Subsequently, a rapid and sustained phosphorylation of the pro-apoptotic players, the nonreceptor protein tyrosine kinase Src, c-Jun NH_2_-terminal kinase (JNK) and p38 MAPK, triggers the release of mitochondrial cytochrome c and activates the regulatory caspase 9 and the downstream effector caspase 3 of the intrinsic apoptotic pathway [93,95]. The endogenous substrate of caspase 3, PARP, is then cleaved, causing cell death [95]. Protein kinase C-δ (PKC-δ), which is also cleaved and activated upon aplidin exposure, plays a pivotal role in amplifying the apoptotic machinery [95]. Aplidin’s effect is Fas CD95 cell death receptor-dependent in leukemic cells [96]. Most importantly, this drug has anti-angiogenic activities by reducing the secretion of VEGF and the availability of its receptor VEGF R-1 [97,98,99]. Aplidin also exerts its action at the cell membrane where it causes direct oxidative damage and Rac-1 activation, a small GTPase that plays an important role in JNK activation [94,100].

Recently, the tumor suppressor protein p27 was shown to affect the level of cytotoxicity of aplidin [100]. High levels of the p27 protein were found to decrease the sensitivity of the cells to the anti-tumorigenic drug aplidin [100]. Indeed, p27 is a member of the CDK inhibitor family that associates to and inhibits the activity of the cyclin-CDK complex under stress conditions, causing G_1_and G_2_/M arrest [100]. Oxidative stress activates p38 MAPK and JNK which in turn lead to the stabilization of p27 [100]. High levels of p27 result in the inhibition of its ubiquitination and degradation by Skp2 as well as in mitotic blockade. Aplidin, as most anti-cancer drugs, preferentially targets rapidly proliferating cells, thus cells that lack the tumor suppressor gene p27 are more sensitive to the cytotoxic effects of the drug [100]. Importantly, aplidin lacks cross-resistance with other anti-cancer drugs, which renders it suitable for combination therapy [100].

### 4.2. Trabectedin: A Unique Alkylating Agent

**Figure 15:**
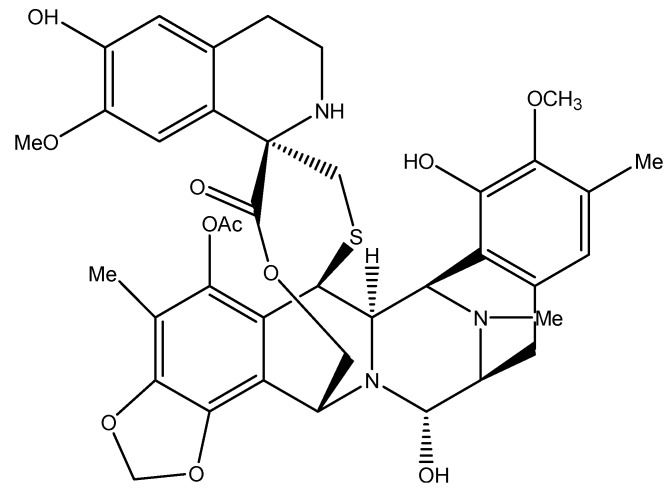
Chemical structure of tunicate-derived drug trabectedin.

The tetrahydroisoquinoline alkaloid trabectedin (ET 743, Figure 15), originally isolated from the marine ascidian *Ecteinascidia turbinate*, is one of the few marine-derived drugs that have reached cancer clinical trials. Currently, it is in phase II clinical trials for the treatment of soft tissue carcinoma, breast, ovarian, endometrial, non-small-cell lung, and prostate cancers [101]. The unique mechanism of action of ET 743 was the reason for its selection for clinical development. This alkylating agent differs from common DNA-damaging agents in the way it binds to DNA, and in its interaction with DNA repair processes, regulation of transcription, and induction of microenvironment changes [102].

Trabectedin possesses three fused tetrahydroisoquinoline rings A, B, and C. The carbinolamine moiety of ring A binds to the exocyclic N_2_ amino group of guanines in the DNA minor groove which diverge from traditional alkylating agents that normally bind to the N7 or O6 in the major groove of the DNA [103]. The adduct formed is an inter-strand crosslink stabilized via van der Waals forces and hydrogen bonding between rings A and B and the neighboring nucleotides [103].

Although ring C does not play a part in DNA binding, it projects out of the DNA and interacts with DNA repair proteins and/or transcription factors. The repair nuclease Rad13 is recruited to the damaged DNA site as part of the transcription-coupled nucleotide excision repair (TC-NER) and forms a Rad13-DNA-trabectedin ternary complex with ring C forming a hydrogen bond with an arginine residue of Rad13 [104]. The formation of this stable complex results in DNA strand breaks that ultimately lead to cell death [104]. Furthermore, cells deficient in homologous recombination (HR), which mediates the repair of DNA double strand breaks, are more sensitive to ET 743 treatment and undergo cell cycle arrest at the S phase followed by apoptosis [105,106].

While trabectedin is a DNA minor groove binder, it can effectively regulate the transcription of genes that bind on the minor groove and genes that bind on the major groove [107,108,109,110,111]. It also induces the breakdown of RNA polymerase II in cells with normal NER [112].

The tumor microenvironment is also affected by ET 743. Studies have shown that this drug causes a decrease in the normal cells’ production of the proinflammatory chemokine (C-C motif) ligand 2 (CCL2), which recruits monocytes to the tumor sites, and a decrease of interleukin-6 (a growth factor implicated in several malignancies), and VEGF. This was observed in both ovarian cancer and myxoid liposarcoma cells [113,114].

Trabectedin is currently approved in Europe as an anti-cancer drug [115]. It is being synthesized and used in combination with doxorubicin to treat ovarian cancer [102,115]. Marketing approval of trabectedin was obtained in 2007 for the treatment of soft tissue sarcoma after failure of the anti-cancer drugs anthracyclines and ifosfamide [102].

### 4.3. Lamellarin D: Overcoming Chemotherapy Resistance

**Figure 16:**
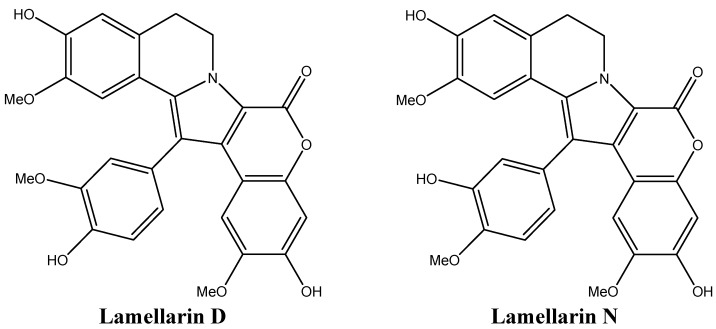
Chemical structures of lamellarins D and N.

The large family of the lamellarins includes the hexacyclic marine pyrrole alkaloid lamellarin D (Lam-D, Figure 16), a molecule with a unique mechanism of action. Lam-D is a potent TOP1 inhibitor that has proven to be effective in treating multi-drug resistant tumor cell lines [116]. Lam-D forms a stable and irreversible complex with TOP1, inhibiting the religation process [116]. The selectivity of Lam-D to malignant cells resides in the difficulty of cells treated with this drug to endure DNA damage [116]. Human prostate cancer cells and leukemic cells were found to be the most susceptible to Lam-D cytotoxicity [117,118]. Most importantly, unlike the TOP2 inhibitor camptothecin, Lam-D is not recognized by Pgp as a substrate and thus is not actively transported out of the cytoplasm [116]. Furthermore, Lam-D induces apoptosis by disrupting the mitochondrial transmembrane potential, leading to the release of cytochrome c to the cytoplasm and activation of Bax, pro-caspase 3, and apoptosis-inducing factor 9 (AIF) [119,120]. The disruption of mitochondrial membrane potential is known to generate reactive oxygen species, which favors the apoptotic response by translocating AIF to the nucleus, increasing the chemosensitivity of non-small cell lung cancer cell lines [120].

Some lamellarins, like lamellarin N (Figure 16) and D, can inhibit protein kinases, mainly CDKs, dual specificity tyrosine phosphorylation activated kinase 1A, casein kinase 1, glycogen synthase kinase-3 and proto-oncogene serine/threonine-protein kinase PIM-1 [121]. Lamellarin N also causes the stabilization of p53, p21, and PARP resulting in increased apoptotic response in cancer cells [121].

### 4.4. Ascididemin: Topoisomerase II Inhibition

**Figure 17:**
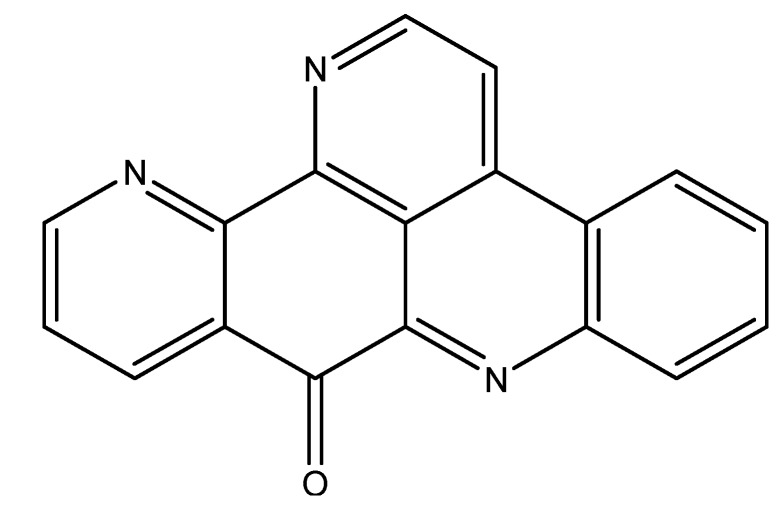
Chemical structure of ascididemin.

The pentacyclic aromatic alkaloid ascididemin (ASC, Figure 17), isolated from the Mediterranean ascidian *Cystodytes dellechiajei*, is a potent cytotoxic anti-cancer agent [122]. The drug profile exhibited by ASC rendered it interesting for further mechanistic studies [122]. This DNA intercalating agent exerts its apoptotic effect via targeting TOP2 and inhibiting its catalytic activity in cancer cells [122]. However, it has little effect on TOP1 and rather hinders the mutated form of TOP1 which is present in camptothecin-resistant tumor cells [122]. Unfortunately, ASC was later shown not to be safe for clinical use [122].

### 4.5. Lissoclinidine B: Stabilization of p53

**Figure 18:**
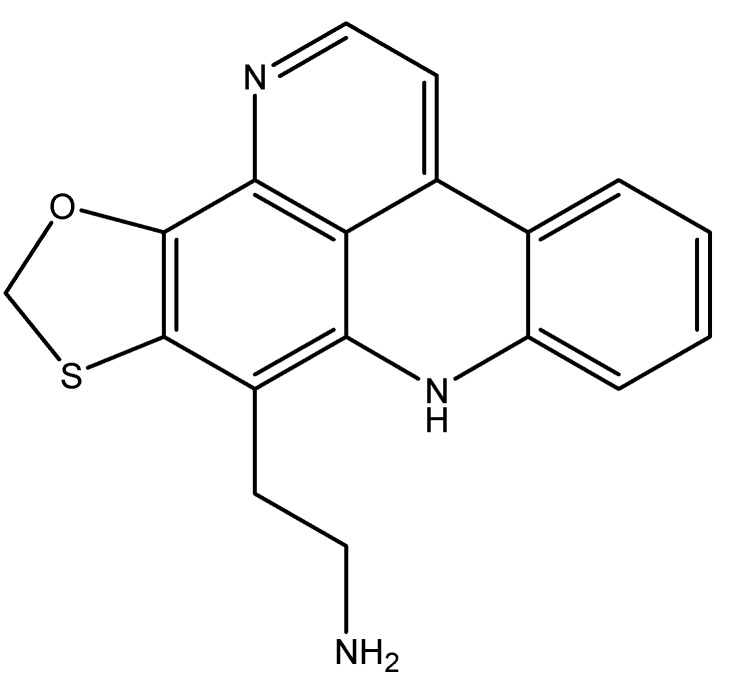
Chemical structure of lissoclinidine B.

The tumor suppressor gene p53 is also termed the guardian of the genome for its central role in preventing the transformation of normal cells into cancer cells [123]. However, p53 is subjected to negative regulation through cellular ubiquitylation and proteosomal degradation by several regulators including human double minute 2 (hmd2). A successful novel therapeutic anti-cancer approach consists of restoring the activity of p53 via targeting its negative modulators [123]. Lissoclinidine B, isolissoclinotoxin B, and diplamine B (Figure 18) are three alkaloids that were isolated from *Lissoclinum badium*. However, only Lissoclinidine B was found to inhibit p53 degradation and to cause cell death in p53 wild-type cancer cells [124].

### 4.6. Polycarpines: p53-Dependent Apoptosis

**Figure 19:**
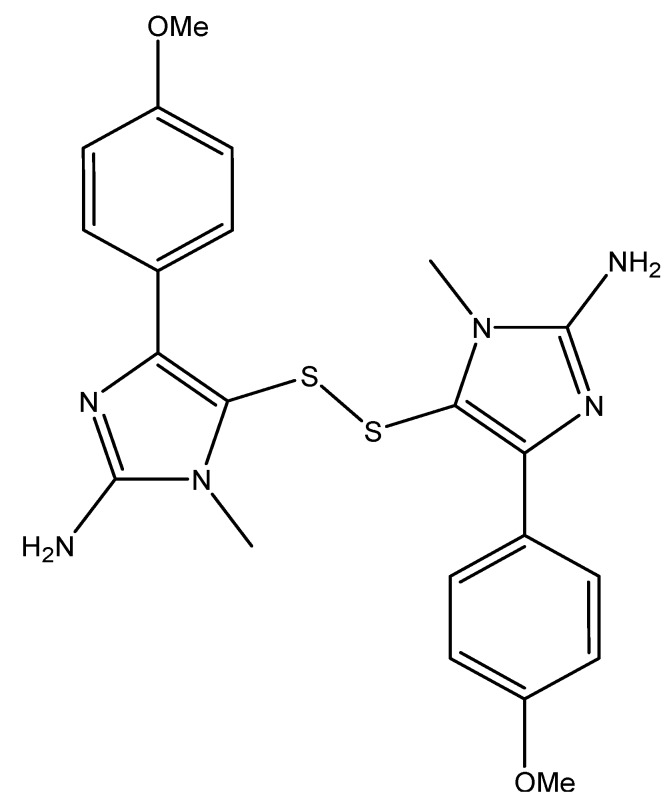
Chemical structure of polycarpine.

The polycarpines (Figure 19) are sulfur-containing alkaloids that were isolated from the ascidian *Polycarpa aurata* [125]. These alkaloids were cytotoxic against a variety of human cancer cells including central nervous system, colon, melanoma, and leukemia [125]. Both polycarpine and its synthetic derivative dimethylpolycarpine were found to induce apoptosis in the JB6 cells in a p53-and caspase 3-dependent manner [125]. This induction was found to be time- and dose-dependent and involved the phosphorylation of p53 at serine 15 by JNKs [125].

### 4.7. Granulatimide and Isogranulatimide: G_2_ Checkpoint Inhibition

**Figure 20:**
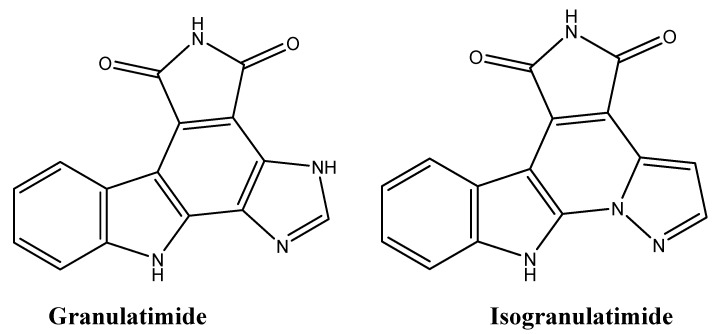
Chemical structures of granulatimide and isogranulatimide.

The first throughput assay screening for G_2_ checkpoint inhibitors led to the isolation of granulatimide and isogranulatimide (Figure 20) from the ascidian *Didemnin granulatum* by bioassay guided fractionation [126]. The advantage of using these G_2_ inhibitors in combination with DNA-damaging agents is that they prevent DNA damage repair in wildtype p53-cancer cell lines, subsequently causing cell death [126].

### 4.8. Cytotoxic Tunicate-Derived Alkaloids with Undetermined Mechanisms of Action

Many tunicate-derived alkaloids were screened for their anti-cancer effects. These bioactive alkaloids are summarized in Table 2 along with their origin and the tumor cell lines they affect.

**Table 2:** Cytotoxic anti-cancer tunicate-derived alkaloids.

Alkaloid	Organism	Cell Lines	IC_50_(μg/mL)	References
Coproverdine	Tunicate	P388	0.95	[127]
Eudistomins	*Eudistoma gilboverde*	LOX, OVCAR-3, COLO-205, MOLT-4	<1.0	[128]
Haouamine A	*Aplidium haouarianum*	HT-29	0.1	[129]
Haterumaimide F	*Lissoclinum voeltzkowi*	P388	0.0055	[130]
Kottamides A-D	*Pycnoclavella kottae*	P388	>10	[131]
Perophoramidine	*Perophora namei*	HCT116	60	[132]
Pibocin B	*Eudistoma* sp.	Ehrlich carcinoma cells	Not determined	[133]
Sebastianines A and B	*Cystodytes dellechiajei*	HCT116	<10	[134]
Sulcatin	*Microcosmus vulgaris*	J774	<10	[135]

## 5. Anti-Cancer Alkaloids Derived from Algae: an Untapped Area of Study

**Figure 21:**
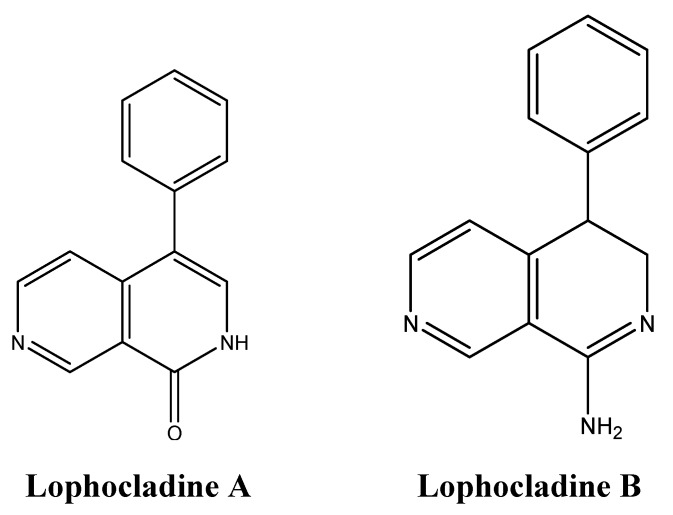
Chemical structure of lophocladines A and B.

Although marine organisms are rich in alkaloids, few marine algae were found to produce anti-cancer secondary metabolites belonging to this class of compounds [136]. The red alga *Lophocladia* sp. produces lophocladines A and B (Figure 21) that were tested for their cytotoxic effects [137]. While lophocladine A was inactive against NCI-H460 lung cancer and neuro-2a neuroblastoma, lophocladine B exhibited moderate cytotoxicity against MDA-MB-435 and NCI-H460 lung cancer cells [137]. Similar to Dolastatin 10, symplostatin 1 and vinblastine, lophacladine B induces morphological changes in treated cells due to complete depolymerization of microtubules, but does not affect the actin microfilaments and causes G_2_/M mitotic cell cycle arrest [137].

## 6. Conclusions

The possibility of medicinal breakthrough discoveries from the marine world has radically increased in the last few years and paralleled the advances in biotechnology. However, with no ethno-medical history to base our search on, the quest for anti-cancer marine alkaloids and the discoveries made until now in this field are quite striking, especially when considering the short exploration time. Significant efforts were undertaken to isolate these bioactive secondary metabolites from marine flora and fauna frequently via bioassay guided fractionation. Cyanobacteria, fungi, sponges, algae, and tunicates have proven to be a source of a large array of alkaloids, many of which can potentially compete with anti-cancer alkaloids derived from plants and which are in clinical trials or in clinical use. Although the majority of these secondary alkaloids were identified in cyanobacteria, the most studied ones are from sponges and tunicates. Alkaloids derived from sponges and tunicates have various anti-cancer activities including anti-angiogenic, anti-proliferative, topoisomerase inhibition, tubulin disruption and apoptosis inducers. Two alkaloids derived from tunicates, aplidin and trabectedin, are already in phase II cancer clinical trials.

Several modes of action of marine-derived alkaloids are similar to those of their terrestrial counterparts while others have completely novel mechanisms (Table 3). The complementarity among drugs with different modes of action is crucial for combination therapy. Indeed the use of trabectedin in combination with pegylated liposomal doxorubicin has already been approved and was shown to increase the progression-free survival of patients with relapsed platinum-sensitive ovarian cancer [115]. In screening studies, the combination of G_2_ checkpoint inhibitors with DNA damaging agents, which synergize to prevent DNA repair mechanisms, has resulted in further sensitizing cancer cells and enhancing cell death [126].

**Table 3:** Mechanisms of apoptosis induction by marine alkaloids.

Alkaloid	Organism	Mechanisms of Action	References
Apratoxin	Cyanobacteria	G_1_ cell cycle arrest, inhibition of FGFR	[138]
Hectochlorin	Cyanobacteria	Hyperpolymerization of actin filaments	[10,11]
Largazole	Cyanobacteria	HDAC inhibitor	[20,21]
Lyngyabellin	Cyanobacteria	Hyperpolymerization of actin filaments	[14]
Shearinine E	Fungi	Inhibition of EGF	[25]
Aaptamine	Sponges	↑p27, G_2_/M cell cycle arrest	[66]
Aldisine alkaloids	Sponges	Inhibition of MEK-1, CDK1, Raf/MEK/MAPK	[67,68]
Bastadin 6	Sponges	Inhibition of VEGF and bFGF	[72]
E7974	Sponges	G_2_/M cell cycle arrest, cleavage of caspase 3 and PARP, disruption of mitotic spindle formation	[43]
Hemiasterlin	Sponges	Tubulin depolymerization	[40]
Kuanoniamine A	Sponges	G_1_ cell cycle arrest	[70]
Makaluvamines	Sponges	Inhibition of TOP2	[56]
Motuporamine C	Sponges	Inhibition of β1-integrin activation	[75]
Naamidine	Sponges	↑p53, p21 Cdk↑, cleavage of capases 3, 8 and 9 and PARP	[61]
Neoamphimedine	Sponges	Inhibition of TOP2	[49]
Renieramycin M	Sponges	↑p53, Bcl-2↓, Mcl-1↓, Sensitization of cells to anoikis	[57]
Spermatinamine	Sponges	Inhibition of isoprenylcysteine carboxyl methyltransferase	[76]
Variolin B	Sponges	G_1_ and G_2_ cell cycle arrest	[139]
Aplidin	Tunicates	↑p27, G_1_ cell cycle arrest, ↑ROS, ↑Src, ↑JNK, ↑p38MAPK, cytochrome c release, cleavage of caspases 3 and 9, PARP cleavage, ↓VEGF	[93,99,100]
Ascididemin	Tunicates	Inhibition of TOP2	[122]
Granulatimide	Tunicates	Inhibition of G_2_ checkpoint	[126]
Lamellarin D	Tunicates	Inhibition of TOP1 and TOP2, cytochrome c release, cleavage of caspases 3 and 9, AIF translocation to the nucleus, ↑BAX	[116,120,121]
Lissoclinidine B	Tunicates	Inhibition of hdm2	[123]
Polycarpines	Tunicates	↑p53	[125]
Trabectedin	Tunicates	DNA alkylation, S cell cycle arrest, RNA pol II breakdown, ↑CCL2, ↓VEGF, ↓IL-6	[102]

The search for new drugs with improved profiles is imminent for cancer treatment. Despite the recent advances in drug discovery from marine organisms, the marine environment is still to be considered a relatively untapped resource particularly for discovering promising anti-cancer drugs. There is no doubt that the marine organisms, which have had to endure predation and stressful habitats over prolonged years of evolution, hold great promise for the future of anti-cancer drug discovery.
